# Study protocol: randomized controlled trial of manualized components in home visitation to reduce mothers’ risk for child maltreatment

**DOI:** 10.1186/s12889-020-8237-4

**Published:** 2020-01-30

**Authors:** Merel de Wit, Patty Leijten, Claudia van der Put, Jessica Asscher, Merian Bouwmeester-Landweer, Maja Deković

**Affiliations:** 1 Research Institute of Child Development and Education, University of Amsterdam, PO Box 15780, 1001 NG Amsterdam, the Netherlands; 2 Clinical Child and Family Studies, Utrecht University, PO Box 80125, 3508 TC Utrecht, the Netherlands; 3 The Netherlands Center for Preventive Youth Health (NCJ), Churchilllaan 11, 3527 GV Utrecht, the Netherlands

**Keywords:** Child maltreatment, Home visitation, Program components, Manualized program, Prevention

## Abstract

**Background:**

This study tests whether home visitation to prevent child maltreatment can be improved by adding manualized program components, targeting four key risk factors for child maltreatment: low parental self-efficacy, high levels of perceived stress, parental anger, and post-traumatic stress symptoms. Home visitation is widely implemented, but effects on child maltreatment risk tend to be modest at best. Home visitation tends to be rather flexible (i.e., professionals decide how to support each family). We will test whether adding manualized program components increases program effectiveness, by ensuring that key risk factors are addressed, while maintaining flexibility. In addition, we will test whether any component effects on reduced child maltreatment risk can be explained (i.e., is mediated) by ameliorated risk factors. Lastly, we will test whether the components are more effective for some mothers (e.g., those at highest child maltreatment risk) than for others.

**Methods:**

We will conduct a randomized controlled trial among 398 mothers enrolled in a Dutch home visiting program targeting families at risk for child maltreatment. Mothers in the experimental group will receive the manualized components in two consecutive home visits, while mothers in the control group will receive regular home visits (care as usual). Mothers will fill out questionnaires at four time points: before and after each of the two home visits. Outcome variables include the four targeted risk factors parental self-efficacy, perceived stress, parental anger, and (recognition of) post-traumatic stress symptoms, as well as parenting practices (e.g., rejection and affection), and risk for child maltreatment.

**Discussion:**

This study aims to determine whether adding manualized program components to a flexible home visiting program increases program effectiveness on risk for child maltreatment. In addition, our test of whether the effects of the components on risk for child maltreatment is explained (i.e., mediated) by amelioration of the targeted risk factors, may contribute to our understanding of the role of these risk factors in child maltreatment. Our tests of which mothers benefit most from adding the components may help move the field towards evidence-based personalized family support.

**Trial registration:**

This trial has been retrospectively registered in the Netherlands Trial Register (NL8005).

## Background

Child maltreatment comes with serious long-lasting consequences for its victims, including physical and mental health problems, and poor academic and employment outcomes [1–5]. Home visiting programs are among the most widely implemented programs for the prevention of child maltreatment [6, 7]. Yet, these programs tend to yield only modest effects on reduced risk for child maltreatment, on average around Cohen’s *d* = 0.24–0.29 [8–10]. This means that of one hundred families receiving home visitation, only seven to nine actually benefit more from such programs than from care as usual [11]. Although it is common for prevention programs to yield relatively small effects [10, 12], because only a subset of the families will develop towards child maltreatment, these numbers highlight the need to increase the effectiveness of home visiting programs to prevent child maltreatment.

On average, home visiting programs are less manualized than other parenting programs (e.g., parenting group programs) [13]. As such, they allow for flexibility – professionals can decide how to support each family, based on clinical experience and perceived individual family needs [14]. Many scholars argue for this flexible approach [15, 16]. Other scholars, however, argue for manualized programs, based on scientific evidence of effective ways to address key risk factors to increase the likelihood of program effectiveness [17, 18]. Although these standpoints may seem incompatible, manualizing a program does not necessarily have to compromise the flexibility that professionals have in delivering the program [19]. Adding a limited set of manualized components that target key risk factors to flexible, largely non-manualized home visitation, may increase program effectiveness, by ensuring that certain key risk factors are targeted in all families, while allowing professionals to maintain flexibility. For example, professionals can still decide, based on clinical experience and individual family needs, how to organize their sessions (e.g., the content of what they discuss with parents). Indeed, home visiting programs that ensure that specific program content is delivered, for example using fidelity checks, tend to yield larger effects than home visiting programs that do not use such checks [20]. In this experimental study, we will test whether adding manualized components that target four key risk factors increases the effectiveness of a home visiting program to ameliorate these risk factors, and to reduce risk for child maltreatment.

### Risk factors targeted in the current study

We selected four dynamic (i.e., malleable) key risk factors for child maltreatment to explicitly target in a home visiting program to prevent child maltreatment: compromised feelings of parental self-efficacy, high levels of perceived stress, parental anger, and post-traumatic stress symptoms [21–23]. First, lower parental self-efficacy, i.e., the belief to be less able to perform the parenting role successfully and to have less control over a child’s behavior and development, may limit mothers’ ability to persist in parenting practices that take more effort [24, 25]. Mothers who feel less self-efficacious tend to be less warm towards their children and use less positive and sensitive parenting practices [26, 27]. Instead, they are more inclined to engage in harsh and inconsistent parenting practices [27, 28]. Therefore, increasing parental self-efficacy may support mothers in sensitive parenting, reducing the risk for child maltreatment. Meta-analytic data support this hypothesis, by showing that child maltreatment prevention programs that include components to increase parental self-efficacy tend to be more effective in reducing mothers’ risk for child maltreatment than programs without such a component [10].

Second, mothers who perceive high levels of stress experience more mental health problems [29, 30], which can lead to engagement in more intrusive, punitive and harsh parenting practices [31–33]. Increasing mothers’ skills to cope with stress, might help them to relieve their stress, giving them more mental space to adopt positive parenting practices in challenging situations and reducing their risk for child maltreatment. Indeed, a meta-analysis shows that child maltreatment prevention programs that explicitly include components to enhance personal skills (e.g., stress management skills) tend to be more effective in reducing mothers’ risk for child maltreatment than programs without such a component [10].

Third, mothers who have difficulty regulating their anger are more inclined than other mothers to express their anger in ways that are harmful for their children [29, 34]. Anger regulation difficulties may be caused by both the extent to which mothers experience feelings of anger, and the extent to which they express their anger in harmful ways [35]. Feelings of anger in mothers at risk for child maltreatment are often intensified by mothers’ dysfunctional attributions about their child’s behavior [36]. For example, mothers might believe that their child’s challenging behavior is intended to upset or annoy them, which may intensify their feelings of anger. Strong feelings of anger can then make them resort to harmful ways of expressing their anger [36]. Thus, altering parents’ dysfunctional attributions and supporting them to express their anger in non-harmful ways, may help to reduce risk for child maltreatment. Indeed, adding a program component focused on dysfunctional attributions and anger management to a parent group training reduced risk for child maltreatment at termination of the program and reduced long-term dysfunctional attributions [37]. This could also apply to home visiting programs.

Last, mothers at risk for child maltreatment tend to have experienced more traumatic events than the general population, increasing their risk for post-traumatic stress symptoms (e.g., emotional numbness and increased arousal) [38, 39]. These symptoms may hamper mothers’ emotional availability to their children and may make it difficult for mothers to be aware of their own emotions until they are so strong that they resort to harsh and punitive behavior [40, 41], increasing the risk for neglect and aggression [42, 43]. In addition, post-traumatic stress symptoms may interfere with intervention effects and increase the risk for drop-out [38, 39, 44–46] . Adequate recognition of these symptoms and referral by home visitors to professional help may therefore reduce mothers’ risk for child maltreatment [40].

In this study, we test whether manualized components designed to target these four key risk factors for child maltreatment ameliorate these risk factors and whether they improve parenting practices and reduce risk for child maltreatment. To further improve our understanding of the role of these four risk factors in the reduction of risk for child maltreatment (i.e., our theory of change), we will also test whether amelioration of these risk factors explains (i.e., mediates) the effects of the components on reduced risk for child maltreatment.

### Potential differential effects

Not all mothers may benefit equally from these components. On the one hand, mothers who are at highest risk considering the targeted risk factors may benefit more as they have the largest room for improvement [47, 48]. On the other hand, mothers who are at lower risk may benefit more, as they may be more able to engage with program content [49]. Other aspects may also influence the degree to which mothers benefit from the components, such as children’s temperament. For mothers whose child is often frustrated or hard to soothe, it may be more difficult to apply newly learned behaviors (e.g., stay calm when their child upsets them) [50]. However, these mothers may be in greater need for strategies to deal with this child behavior, and thus benefit more from components that target their stress and anger regulation [51]. Furthermore, the accumulation of life events (e.g., quitting one’s job or death of a family member) may either hinder mothers to benefit from the components, if it makes mothers less able to engage with program content [52], or may increase the effectiveness of the components, if they buffer the adverse effects of accumulation of life events [53]. In this study, we will therefore examine maternal (initial levels of targeted risk factors), child (temperament), and family (life events) characteristics as putative moderators of the effects of the manualized components on the targeted risk factors and on risk for child maltreatment. Knowledge on differential effects of the added components can serve to guide personalization of programs. In other words, it can support home visiting programs in their goal to serve individual family needs in an evidence based way.

### Study aims

In this study, we will test (1) whether manualized components designed to target four key risk factors for child maltreatment (low parental self-efficacy, stress, parental anger, and post-traumatic stress symptoms) indeed ameliorate these risk factors; (2) whether adding these manualized components to a home visiting program improves parenting practices and reduces the risk for child maltreatment; (3) whether any effect of the manualized components on reduced risk for child maltreatment can be explained (i.e., is mediated) by amelioration of the four targeted risk factors; (4) whether some mothers benefit more from the manualized components than other mothers in terms of ameliorated risk factors and reduced risk of child maltreatment.

## Methods/design

### Design

We will conduct a randomized controlled trial in the context of an existing Dutch home visiting program aimed at the prevention of child maltreatment (Supportive Parenting, in Dutch “*Stevig Ouderschap*” [54]). Mothers will be randomly assigned to receive either two consecutive home visits that include the additional manualized components (i.e., manualized home visiting), or to receive standard home visits (i.e., care as usual) with a 1:1 allocation using a computerized random number generator.

### Participants

Participants will be *N* = 398 mothers enrolled in the Supportive Parenting program. The program is part of care as usual in many municipalities in the Netherlands. Approximately 51% of all Dutch newborns live in a municipality that offers Supportive Parenting. Their parents receive a screening questionnaire in the first week after giving birth (the Instrument for early identification of Parents At Risk for child Abuse and Neglect; IPARAN [49]). Approximately 6.5% of families show an elevated risk on child maltreatment based on this instrument, for example, due to a history of child maltreatment, or a lack of social support in either one or both of the parents. These families are offered Supportive Parenting. Supportive Parenting targets both mothers and fathers. That said, mothers are mostly present during Supportive Parenting home visits [55] and we therefore focus specifically on mothers in this study.

### Intervention

The Supportive Parenting program consists of six 90 min home visits conducted by child health nurses within the first 18 months after birth. During these visits, nurses support parents by enhancing their parenting knowledge and skills and by strengthening their social support system. A large part of every home visit is flexible and parents can choose the topics they wish to discuss (for a more detailed description of the program, see [54, 56]). The nurses who deliver the program work in regional youth health care centers, similar to Well Baby clinics in the United States, and have followed a two-day training for delivering the Supportive Parenting program. A previous study among 469 mothers shows that mothers who received Supportive Parenting report more appropriate expectations of their children, less oppression of their child’s independence, and less worrisome child development compared to mothers receiving care as usual. Regarding risk for child maltreatment, findings were mixed. There was no mean difference in risk for child maltreatment, but a higher percentage of mothers who received Supportive Parenting showed a reliable reduction in risk for child maltreatment (22%), relative to mother receiving care as usual (8%) [57].

### Added Manualized components

We designed four manualized components targeting low parental self-efficacy, high levels of perceived stress, parental anger, and post-traumatic stress symptoms**.** Nurses will implement these components in two consecutive home visits for mothers in the experimental group.

#### Increasing parental self-efficacy

Nurses will give structured positive feedback about mothers’ parenting practices at least twice in both home visits. Nurses are free to choose which parenting practices they will target in their feedback, but the feedback is designed to tap into multiple sources of self-efficacy: mothers will have a sense of mastery, they will be verbally persuaded, and they will be compared positively to other mothers [24]. This component is based on the work of Mouton and Roskam [58], who showed that a similar systematic way of providing positive feedback to mothers increased parental self-efficacy and the use of positive parenting practices.

#### Reducing levels of perceived stress

Nurses will provide mothers with a 10-min guided imagery relaxation exercise, on audio, in which mothers are instructed to imagine a place where they feel safe, calm, and relaxed. The script used in the exercise is based on the work of Naparstek [59] and the Dartmouth Student Wellness Center [60]. We adapted the wording and some of the examples to suit mothers with low educational levels, using specific guidelines for Dutch texts [61], and the input of several nurses who work as home visitors for Supportive Parenting. Guided imagery relaxation exercises like these can positively affect both perceived stress and physiological stress in a range of populations (e.g., [23, 62, 63]). Listening to guided imagery exercises has also shown to decrease levels of anxiety and pain in hospital settings, for example prior to surgery [64]. Nurses will encourage mothers to listen to the exercise daily by playing the audio file during the home visit, explaining the positive effects it may have for their well-being, and helping mothers choose a time and place for the daily exercise.

#### Reducing parental anger

This module is designed to alter negative attributions that mothers might have about their child’s behavior and provide mothers with alternative strategies to respond to child behavior that triggers anger. To do this, nurses will discuss several ways to recognize anger and techniques to calm down when feeling angry. More specifically, nurses will discuss mothers’ anger triggers (i.e., child behavior that evokes anger in mother) and negative attributions (e.g., ‘my child tries to upset me’) using a set of cards, based on the work of Kock and colleagues [21]. Each card describes child behavior that may evoke anger in mothers, possible reasons for the child’s behavior and possible strategies to respond to such behavior.

#### Recognizing post-traumatic stress symptoms

Nurses will use the two-item version of the abbreviated Post Traumatic Stress Disorder Checklist – Civilian (abbreviated PCL-C [65]) to assess the level of mothers’ post-traumatic stress symptoms. This checklist is an adequate screening instrument for post-traumatic stress disorder (PTSD) with a sensitivity of .95 and a specificity of .50 in women [65]. Nurses will motivate mothers who score above the threshold, indicating that they might suffer from PTSD, to consult their general practitioner in order to receive therapy (e.g., eye movement desensitization and reprocessing therapy; EMDR [66, 67]).

### Procedure

**Fig. 1:**
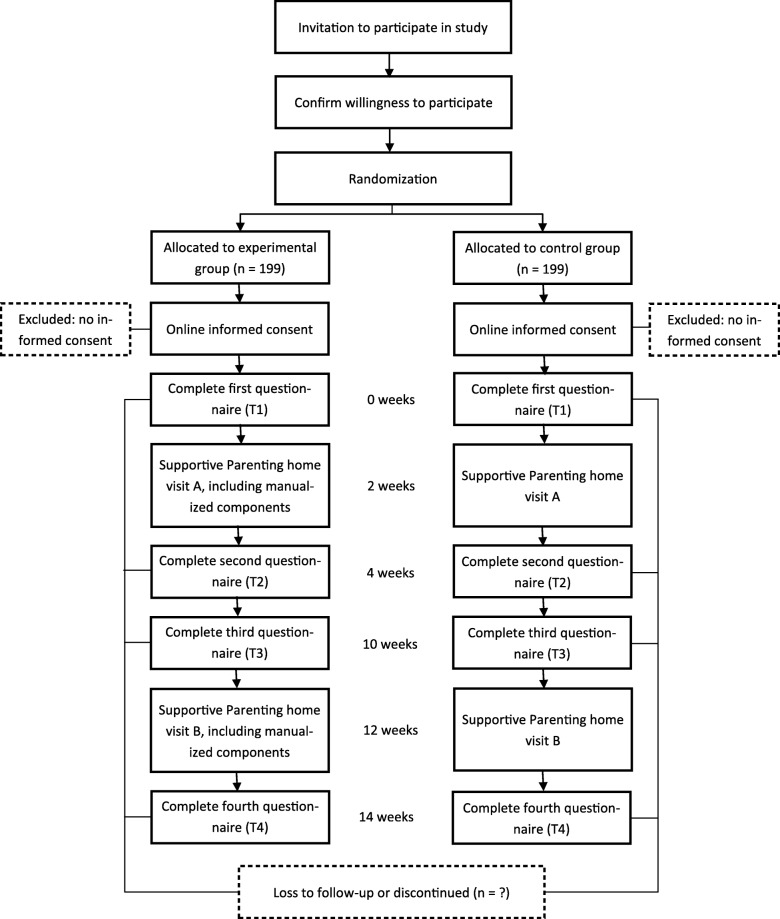
Participant flow chart

Figure 1 shows an overview of the study procedure. Nurses will inform mothers enrolled in Supportive Parenting about the study purposes and invite them to participate. To support nurses in this, we will contact each of them monthly, by phone, email, or in person. Mothers interested in participating send their contact details to the research team, who will contact them to discuss the study procedures and to sign informed consent for study participation. See the Appendix for the consent form used in this study. Only after informed consent is obtained after explanation by the research team, participants will be allocated to either the experimental or the control group by the researchers, using a computerized random number generator. Participants are not blind to the conditions (they know whether they receive added components or not), but mothers are not aware of the study hypotheses.

This study will follow mothers over the course of any two consecutive Supportive Parenting home visits, which will be named home visit A and B in this study. Home visits tend to be about three months apart from each other, but this may vary in clinical practice. Mothers will fill out online questionnaires four times; one to two weeks before home visit A visit (T1), one to two weeks after home visit A (T2), one to two weeks before home visit B (T3), and one to two weeks after home visit B (T4). We offer mothers assistance in filling out the questionnaires by phone. We will send text messages by phone to remind mothers to fill out the questionnaires. Mothers receive a compensation of 5 euros for each questionnaire they fill out, and an additional 5 euros if they fill out all the questionnaires.

Mothers in the experimental group will receive two Supportive Parenting home visits (A and B) in which nurses will deliver the manualized components. All four components will be delivered in both home visits. Mothers in the control group will receive two regular Supportive Parenting home visits that do not include the manualized components. As we do not expect any adverse effects from these components, we did not set any criteria to discontinue the components. There are no restrictions for mothers to seek any additional help, but mothers are asked to report it if they do so.

This study has been approved by the Ethics Review Board of the Faculty of Social and Behavioral Sciences of the University of Amsterdam (ref nr: 2018-CDE-9258) and the trial has been registered in the Dutch Trial Register (NL8005). Any meaningful changes will be reviewed by the Ethics Review Board of the Faculty of Social and Behavioral Sciences of the University of Amsterdam and will be reported in the effect paper.

### Fidelity

All nurses will receive a half-day training to deliver the manualized program components, in addition to the training they already received to deliver Supportive Parenting. Nurses will be explicitly instructed, and repeatedly reminded in follow-up contact, to deliver the manualized components to mothers in the experimental group, and not to mothers in the control group. To monitor fidelity of the manualized components, and detect any possible contamination between the two groups (i.e., nurses might unwittingly offer some aspects of the components to mothers in the control group as well), nurses will complete a checklist after each home visit in which they specify to what extent and in what way the four risk factors have been targeted. This will be done for all home visits to all mothers (i.e., experimental and control group).

As an additional fidelity check, we will include a second control group, consisting of 25 mothers in one of the regions that offer Supportive Parenting, but where nurses have not yet received training in the four manualized components. This is a non-randomized group, because all participating mothers in this region will be allocated to this second control group. Mothers and nurses in this group will fill out the same questionnaires and checklists as mothers and nurses included in the main part of the study. Comparing the scores of the two control groups allows us to estimate to what extent training nurses in the use of the manualized components unwittingly impacted mothers in the control group (i.e., contamination). Should we detect contamination, as indicated by the checklists, and trends that the regular control group outperforms the non-randomized control group, this could suggest that the effects of adding manualized components are larger than measured in this study.

### Measures

Table 1 provides an overview of the study variables and their assessment moments. We adapted the wording of some items from the original questionnaires to make them suitable for mothers with low educational levels, using specific guidelines for Dutch texts [61]. Following these guidelines, we tried to keep items short, use an active voice, and avoid using difficult words. For example the phrase ‘I do not do things that I know my child wants me to do,’ was rephrased as ‘I do not do what my child wants me to do.’ With the exception of life events, mothers report on all measures about the last month.

**Table 1:** Overview of Study Variables

Outcome	Measure	T1	T2	T3	T4	Reference
Primary outcomes						
Parental self-efficacy	Parenting Stress Index	x	x	x	x	[68]
Levels of perceived stress	Perceived Stress Scale	x	x	x	x	[69]
Parental anger	Parental Anger Scale	x	x	x	x	[70]
Post-traumatic stress symptoms	Post-Traumatic Stress Disorder Checklist – Civilian	x	x	x	x	[65]
Secondary outcomes						
Risk for child maltreatment	Instrument for early identification of Parents At Risk for child Abuse and Neglect	x			x	[54]
Parenting (Rejection, Hostility, Attention, Affection)	Comprehensive Parenting Behavior Questionnaire	x			x	[71]
Potential moderators						
Child temperament (Soothability, Negative emotionality)	Revised Infant Behavior Questionnaire	x				[72]
	Very Short Form of the Revised Infant Behavior Questionnaire					[73]
Life events	Parenting Stress Index	x^a^			x^b^	[68]

*Note.* ^a^ = life events experienced in the past 12 months, ^b^ = life events experienced during the study

### Primary outcome measures

#### Parental self-efficacy

Mothers report on feelings of self-efficacy on the shortened Sense of Competence subscale of the Parenting Stress Index [68]. Mothers rate nine items, such as ‘I feel that I am not very good at being a parent’ on a six-point scale ranging from 1 (*I totally disagree*) to 6 (*I totally agree*). All items are reverse coded such that higher scores indicate higher levels of parental self-efficacy.

#### Levels of perceived stress

Mothers report on feelings of stress on the ten-item-version of the Perceived Stress Scale (PSS-10 [69]). This is a widely-used questionnaire that assesses the degree to which respondents experience situations in one’s life as stressful, that is, unpredictable, uncontrollable and overwhelming, in the past month. The internal consistency of the PSS-10 varies between α = .74 and α = .91 [74]. Mothers rate ten items, such as ‘In the last month, how often have you found that you could not cope with all the things that you had to do?,’ on a five-point scale ranging from 1 (*never*) to 5 (*very often*). Four items are reverse coded such that higher scores indicate higher levels of perceived stress.

#### Parental anger

Mothers report on parental anger on the ‘expression’-subscale of the Parent Anger Scale (PAS [70]). This questionnaire assesses anger experience in the parent-child context. The PAS expression subscale has high internal consistency (α = .95) and correlates to other measures of negative affect and discipline strategies [75]. Mothers rate 11 items such as ‘I get so angry with my child, that I scream or yell at my child’ on a seven-point scale ranging from 0 (*never*) to 6 (*several times a day*).

#### Post-traumatic stress symptoms

Mothers report on post-traumatic stress symptoms on the six-item version of the abbreviated PTSD checklist – civilian (abbreviated PCL-C [65]). This is a more extensive version of the checklist that nurses will use as a screening tool in the manualized component on recognition of post-traumatic stress symptoms. The abbreviated PCL-C is an adequate screening instrument for PTSD, with a sensitivity of .95 and a specificity of .57 in women [65]. Mothers rate the extent to which six symptoms, such as ‘repeated, disturbing memories, thoughts or images of the stressful experience,’ occurred on a five-point scale ranging from 0 (*not at all*) to 4 (*extremely*).

In order to measure nurses’ ability to recognize post-traumatic stress symptoms in mothers, nurses report to what extent they think a mother suffers from post-traumatic stress symptoms on a five-point scale ranging from 0 (*not at all*) to 4 (*extremely*). We will correlate mothers’ and nurses’ answers with each other as an indication of how adequately nurses recognize post-traumatic stress symptoms in mothers.

### Secondary outcome measures

#### Parenting practices

Mothers report on four dimensions of parenting practices on the following subscales of the Comprehensive Parenting Behavior Questionnaire (CPBQ [71]): Rejection (2 items, such as ‘Sometimes I am really fed up with my child, and this clearly shows’); Hostility (4 items, such as ‘Sometimes I can be harsh when my child is really annoying’); Attention (4 items, such as ‘I regularly play or talk with my child for at least 5 min, with our attention focused on one other, just for fun’); and Affection (4 items, such as ‘I often cuddle my child’). All items are rated on a five-point scale ranging from 1 (*totally not applicable*) to 5 (*completely applicable*). In the hostility subscale, one item is reverse-coded such that higher scores indicate higher levels of hostility.

#### Risk for child maltreatment

Mothers report on their risk for child maltreatment on the Instrument for early identification of Parents At Risk for child Abuse and Neglect (IPARAN [54]). This is the same questionnaire that youth health care centers use to screen families for the Supportive Parenting program. Mothers rate nine items, such as ‘I can get so angry that I lose control’ on a scale ranging from 1 (*always*) to 4 (*never*) and answer seven yes/no items, such as ‘I feel that my parents/carers hit me too much as a child.’ The items differ in their scoring and answers correspond with scores of either 0, 0.1, 0.2, 0.3, 0.5, or 1 (for more details, see [76]). The IPARAN has shown to adequately predict future reports of child maltreatment [76]. Youth care centers use a simplified rating system where answers are scored as 0, 0.5, 1, 1.5, or 2. Families where at least one parent scores ≥3 are offered the Supportive Parenting program.

### Potential moderators

#### Child’s temperament

Mothers report on two dimensions of their child’s temperament, i.e., soothability and negative emotionality. Mothers rate child soothability on 18 items, such as ‘when singing or talking to your baby, how often did s/he soothe immediately’, from the Revised Infant Behavior Questionnaire (IBQ-R [72]). Half of the items are reverse-coded such that higher scores indicate that children are easier to soothe. The internal consistency of the soothability subscale varies between α = .81 and α = .83 [72]. Mothers rate child negative emotionality on 12 items from the Very Short Form of the IBQ-R (IBQ-R VSF [73]) that were derived from the sadness, distress to limitations, and fear subscales of the original IBQ-R, such as ‘at the end of an exciting day, how often did your baby become tearful.’ The internal consistency of the negative emotionality subscale varies between α = .72 and α = .88 [73].

The original subscales use a seven-point scale ranging from 1 (*never*) to 7 (*always*). However, to accommodate for mothers with low educational levels we use four of the original answer categories for both scales (*almost never, less than half of the time, more than half of the time, almost always*). We chose to use four original response options and leaving three out, rather than combining options, to ensure that scores can be compared to scores in other studies using these measures.

#### Life events

Mothers report how many life events happened to them in the past 12 months and during the study on the ‘life events’-subscale of the Parenting Stress Index (PSI [68]). This subscale consists of thirty yes/no-items such as ‘I got fired/I quit my job’ or ‘I had a miscarriage.’

As most of the questionnaires that we used are copyright protected, we will not publish the data collection forms.

### Protection of data privacy

Data will be stored at secured servers of the University of Amsterdam and will only be accessible for researchers on this project. During the data collection process, participants’ names and contact information will be stored in a separate password-secured file that is only accessible for the researchers directly involved in this study. After data collection is complete, the contact information of participants who give consent to be contacted for future research will be stored in an encrypted file and will only be accessible for the research data manager. Contact information of participants who do not give consent to be contacted for future research will be deleted after data collection is complete.

### Sample size calculation

As we will compare two active conditions, we expect to find relatively small effects. More specifically, we expect moderate effects on the targeted risk factors (i.e., our more proximal outcome measure) and a small effect on mothers’ risk for child maltreatment (i.e., our more distal outcome measure). Previous additive studies (i.e., studies in which effects of an intervention are compared to effects of the same intervention with one or more additional components) also demonstrated small to moderate average effect sizes for interventions with added components [77]. Our sample of *N =* 398, with two groups and one-sided tests at α = .05 provide us with a power (1 – β) of .80 to detect effects of *d* = .25 [78]. Following the guidelines of Fritz and MacKinnon [79], our sample size will also allow for sufficient power to detect mediation effects when using mediation analyses based on bootstrapping.

### Analyses

Prior to analyzing the data, missing data will be handled with multiple imputation. To test whether adding manualized components ameliorates four risk factors (i.e., low parental self-efficacy, high levels of perceived stress, parental anger, and post-traumatic stress symptoms), improves parenting practices, and reduces risk for child maltreatment, we will conduct multivariate analyses of covariance (MANCOVA) and an analysis of covariance (ANCOVA) respectively, with baseline scores (T1) as the covariates, with data of T2, T3, and T4 for the risk factors and data of T4 for parenting practices and risk for child maltreatment as dependent variables.

To test whether amelioration of the targeted risk factors by the added components explains (i.e., mediates) reduced risk for child maltreatment we will conduct a mediation analysis using the PROCESS Macro in SPSS [80]. This macro computes 95% confidence intervals of the indirect effects based on 1000 bootstrap samples. Intervals that do not include 0 indicate a mediation effect.

To test who benefits most from the manualized components we will test whether condition × child, maternal, or family characteristic interaction effects predict ameliorated risk factors and reduced risk for child maltreatment. Putative moderators include mothers’ baseline levels of each of the four risk factors (maternal characteristics), children’s temperament (child characteristic), and the number of life events in the past year and during the study (family characteristic).

We will conduct interim descriptive analyses for our progress annual reports for the study funder. However, no interim analyses that answer the research questions of this study will be conducted. The full protocol, anonymized dataset, and the statistical code that will be used in this study will be available upon motivated request after publication of the results in a peer-reviewed paper.

## Discussion

Given the serious consequences of child maltreatment, and the typically modest effects of frequently used home visiting programs, it is important to know how programs can be improved to support families by reducing their risk for child maltreatment. This study strives to guide efforts to increase the effects of Supportive Parenting in particular, and the effects of child maltreatment prevention programs in general, by studying whether adding components that target compromised feelings of parental self-efficacy, high levels of perceived stress, parental anger, and post-traumatic stress symptoms, contribute to program effectiveness.

Scholars often seem divided in arguing for either manualized or flexible treatments (for an overview, see [81]). In this study, we bridge both approaches by adding a select set of manualized components while maintaining a level of flexibility. Our study sheds light on whether borrowing some of the advantages of either approach – manualized components to target key risk factors for child maltreatment and the flexibility to adjust program content according to clinical experience – yields larger effects than a more flexible approach.

Research on the effects of a limited set of program components helps us unravel the effects of parenting programs, by identifying the merit of discrete program components, above and beyond other components and common elements [82]. Knowledge on the effectiveness of program components can therefore refine theories about mechanisms of change that underlie program effectiveness.

Understanding whether any effects of the four manualized components on reduced risk for child maltreatment are mediated by amelioration in the four targeted risk factors, improves our understanding of the role of these risk factors in occurrences of child maltreatment. If amelioration of one or more of the targeted risk factors indeed explains a stronger reduction in the risk for child maltreatment yielded by manualized home visitation, this strengthens our theory that these risk factors indeed contribute to risk for child maltreatment [83].

### Limitations

We add four manualized components, based on the premise that these four combined components together increase the effectiveness of home visitation. This approach does not allow us to draw conclusions about the effectiveness of each single component. While it may seem plausible that any effects on, for example, ameliorated parental anger are caused by the component that explicitly targets parental anger, we cannot exclude the possibility that other components (e.g., the one that targets stress) also contributed to the amelioration of parental anger. Thus, the present study only allows for conclusions about the causal effects of all four components combined.

We will test the effectiveness of the manualized components in two home visits. While two sessions may seem insufficient to change key risk factors for child maltreatment that tend to be persistent over time [84, 85], we opted for this approach, first, to reflect the clinical reality of home visiting programs such as Supportive Parenting. We designed this study to inform professionals on how to increase the effectiveness of Supportive Parenting, while maintaining its ease of implementation. The second reason for opting for a two-session approach is that evidence accumulates that interventions with a limited number of sessions (sometimes even single sessions) can yield meaningful effects [86, 87].

Home visiting programs to prevent child maltreatment target a vulnerable group of families. Improving the effectiveness of these programs has the potential to prevent an array of negative outcomes for these families. This study strives to inform both clinical practice and child maltreatment theory. The manualized components that we test in our study are relatively easy to implement into home visiting programs, enabling home visiting programs to disseminate knowledge on the effectiveness of adding these components into clinical practice. In addition, our tests of which mothers benefit most from adding these components can help move the field towards evidence-based personalized family support. Lastly, this study aims to improve our understanding of the role of four key risk factors in reducing risk for child maltreatment, which may contribute to more refined child maltreatment theories.

### Study status

Data collection and recruitment of participants is ongoing.

## Data Availability

Not applicable.

## Appendix

Consent form for parents.

Study “Manualized Components in Supportive Parenting”

I confirm that I have read the information letter of this study that I received from my nurse. I understand the information and have had the opportunity to ask questions. My questions are answered. I have had enough time to think about my participation in the study.

I know that my participation is completely voluntary and I know that I can withdraw my participation at any time during the research and until seven days after the last questionnaire.

I am aware that both I and my nurse will fill in questionnaires about my family.

I know that the information from the questionnaires will be used only for this study and will be treated confidentially.

I know that information from the questionnaires will be stored as long as necessary (up to twenty-five years).

I know that the data are processed encrypted. I know that codes will be used instead of names.

I am aware that for any complaints about this study, I can contact the Ethics Committee of the Department Pedagogical and Educational Sciences and Teacher Education of the University of Amsterdam, Prof. dr. Henny Bos; e-mail: H.M.W.Bos@uva.nl; Nieuwe Achtergracht 128, 1018 WS Amsterdam.

I agree with my participation in this study

- I agree, start the study
- I do not agree, I do not want to participate in the study

## Abbreviations

ANCOVA: Analysis of covariance
CPBQ: Comprehensive parenting behavior questionnaire
IBQ-R: Revised Infant behavior questionnaire
IPARAN: Instrument for early identification of parents at risk for child abuse and neglect
MANCOVA: Multivariate analyses of covariance
PAS: Parent anger scale
PCL-C: Post-traumatic stress disorder checklist – civilian
PSS-10: Ten-item-version of the Perceived stress scale
PTSD: Post-traumatic stress disorder
